# Oropharyngeal Squamous Cell Carcinoma Treatment in the Era of Immune Checkpoint Inhibitors

**DOI:** 10.3390/v13071234

**Published:** 2021-06-25

**Authors:** Peter L. Stern, Tina Dalianis

**Affiliations:** 1 Manchester Cancer Research Centre, University of Manchester, Manchester M20 4GJ, UK; 2 Department of Oncology-Pathology, Karolinska Institutet, Bioclinicum J6:20, Karolinska University Hospital, 171 64 Stockholm, Sweden; tina.dalianis@ki.se

**Keywords:** oropharyngeal squamous cell carcinomas (OPSCC), human papillomavirus (HPV), tumour microenvironment (TME), immune checkpoint inhibitors, programmed death receptor-1 (PD-1) and ligand -1(PD-L1), T cell effectors, myeloid-derived suppressor cells (MDSC), macrophages, dendritic cells (DC)

## Abstract

While head and neck squamous cell carcinomas (HNSCC) are marginally decreasing due to the reduction in exposure to the major risk factors, tobacco and alcohol, the incidence of high-risk human papillomavirus (HPV)-positive oropharynx squamous cell carcinomas (OPSCC), especially those in the tonsil and base of tongue subsites, are increasing. Patients with the latter are younger, display a longer overall survival, and show a lower recurrence rate after standard-of-care treatment than those with HPV-negative OPSCC. This may reflect an important role for immune surveillance and control during the natural history of the virally driven tumour development. Immune deviation through acquisition of immune-suppressive factors in the tumour microenvironment (TME) is discussed in relation to treatment response. Understanding how the different immune factors are integrated in the TME battleground offers opportunities for identifying prognostic biomarkers as well as novel therapeutic strategies. OPSCC generally receive surgery or radiotherapy for early-stage tumour treatment, but many patients present with locoregionally advanced disease requiring multimodality therapies which can involve considerable complications. This review focuses on the utilization of newly emerged immune checkpoint inhibitors (PD-1/PD-L1 pathway) for treatment of HNSCC, in particular HPV-positive OPSCC, since they could be less toxic and more efficacious. PD-1/PD-L1 expression in the TME has been extensively investigated as a biomarker of patient response but is yet to provide a really effective means for stratification of treatment. Extensive testing of combinations of therapeutic approaches by types and sequencing will fuel the next evolution of treatment for OPSCC.

## 1. Introduction

Head and neck cancers originating in the oral cavity, larynx, pharynx (hypopharynx, nasopharynx, or oropharynx), and in the sinonasal tract are 90% squamous cell carcinomas (SCC) [1,2,3,4]. This is the eighth most common malignancy worldwide in 2018, and overall survival (OS) for localized cancers is more than 90% for salivary gland and mucosal lip sites but closer to 60% for laryngeal and hypopharyngeal sites [5]. The principal associated risk factors are tobacco use, alcohol consumption, high risk (HR) human papillomavirus (HPV) infection (for OPSCC), and Epstein-Barr virus (EBV) infection (for nasopharyngeal squamous cell carcinoma) [1,2,3,4]. When the upper aerodigestive tract is chronically exposed to these carcinogenic elements, dysplastic lesions can develop in the oropharyngeal mucosa and such premalignant neoplasia is a precursor for malignant tumours. The relative prevalence of these risk factors contributes to the variations in the observed distribution of head and neck squamous cell carcinomas (HNSCC) in different areas of the world [1,2,3,4]. Unsurprisingly, HNSCC are a very heterogenous group of cancers which demonstrate different pathologies, prognoses, and treatment options [5]. Genomic analysis of HNSCC has provided comprehensive lists of associated mutations/genetic changes (single nucleotide, confined insertions/deletions, larger alterations including gains and losses from arms of or entire chromosomes) [2,6]. The picture that emerges for HPV-negative HNSCC is of some key driver somatic gene mutations, with each generally associated with considerably less than a quarter of tumours (with the exception of TP53, which is present in a majority (72%)), which then are manifest on a background of other diverse, including passenger, mutations. Most of the driver events inhibit the action of tumour suppressor functions on the cell cycle (p16INK4A; p53), survival (PTEN), WNT signalling (protocadherinFAT1; LIM domain-containing protein AJUBA; NOTCH1), and epigenetic regulation (histone-lysine N-methyltransferases KMT2D and NSD1). Acquisition of oncogenic action on the cell cycle (G1-S-specific cyclinD1), epidermal growth factor receptor (EGFR), and survival have also been documented in some subsets of HPV-negative HNSCC [2,6]. 

## 2. Oropharyngeal Squamous Cell Carcinomas (OPSCC) 

The incidence of HNSCC located at most oral sites is actually marginally decreasing because of the reduced exposure to the tobacco and alcohol risk factors associated with its development. By contrast, the incidence of OPSCC associated with HR HPV is increasing, particularly in young men, which is most apparent in Northern Europe and North America and believed to be the consequence of oral sexual behaviour [5,7]. For SCC originating in the oropharynx, tonsil, and base of the tongue (OPSCC), HR HPV, principally HPV 16, accounts for >80% of the tumours [8,9]. Importantly, patients with HPV16-positive OPSCC display a longer OS and a lower recurrence rate after SOC treatment than patients with HPV-negative OPSCC [7]. Since HPV-related OPSCC behaves differently as a disease when compared to HPV-negative OPSCC, a separate staging system has been developed [10,11]. The assignment to HPV-positive is frequently inferred from p16 immuno-histochemistry whereby the expression of p16 is constitutively unregulated by HPV oncogene suppression of Rb [12]. This type of measure is not fool-proof and additional, more direct, measures of HPV through nucleic acid detection can significantly improve specificity, but gold-standard detection of hr HPV oncogene RNA is unfortunately not regularly utilised [13]. 

For TNM staging (see Legend Table 1), while the T categories for HPV-positive and HPV-negative OPSCC are similar, a T0 level (unknown primary) category is only used in p16-positive metastatic nodes, where the primary is presumed an OPSCC, and the T4b category has been removed from p16-positive OPSCC. For clinical N staging of p16-positive disease: N1 is ipsilateral nodes (one or multiple), but none larger than 6 cm, N2 is contralateral or bilateral nodes less than 6 cm, and N3 is when nodes are greater than 6 cm. Pathological staging is only available to patients where surgery is possible, and for HPV-related (p16-positive) OPSCC the number of positive nodes between 1 and 4 vs. 5 or more impacts outcome, so this is reflected in pN staging. A summary of the clinical and pathological prognostic stage groups is shown in Table 1 [11].

The natural history of HPV-mediated carcinogenesis in HNSCC is not precisely documented. However, in squamous epithelium of the transformation zone of the cervix, about 20% of hr HPV infections result in dysplastic lesions, and the major risk factor for cancer progression is persistent infection [14]. In cases where the normal viral life cycle is aborted and expression of the viral oncogenes E6 and E7 is elevated (e.g., through integration of the virus in cell genome), cell proliferation is stimulated. This virally driven cellular proliferation is causally linked to malignant transformation through unregulated E7 expression degrading the host cell retinoblastoma pocket proteins (RB), inducing S-phase-related molecular changes, with viral E6 binding to and degrading p53 to prevent apoptosis of unscheduled S-phase entry [15]. This leads to a reduced control of the cell cycle which would usually allow for DNA repair of any genetic errors. Any accumulation of mutations can provide advantage for transformation to high-grade cervical intraepithelial neoplastic lesions [15,16]. 

The productive versus transforming fate of an HPV infection may be dependent on the infected cell type of origin. For example, it has been suggested that specific single-layered epithelial cells of embryonic origin at the cervical squamocolumnar junction constitute highly susceptible cells for transforming infections and the origins of cancers [17]. For OPSCC, no obvious virus-infected premalignant lesions have yet been identified, but it is very likely that the natural history of oncogenesis is similar. As documented for cervical cancer, molecular changes in genes of the PI3K pathway are the most frequent genetic alterations found in HPV-driven HNSCC [6,18]. The PI3K signalling pathway affects translation and transcription of multiple targets that are involved in proliferation, survival, and motility [19]. However, while *PIK3CA* alterations appear more common in HPV-positive HNSCC, these are also detected in HPV-negative OPSCC; oncogenic PI3K signalling seems to constitute a driver for many HNSCC, independent of HPV involvement [6]. Genomic profiling of HPV-positive OPSCC shows evidence of heterogeneity in patterns of altered gene expression [20]. 

## 3. Standard of Care (SOC) for OPSCC

Surgical treatment, especially with recent advances in transoral robotic surgery of OPSCC, can sometimes be curative if the tumour is relatively small, has not spread, and is easily accessible, although these procedures are not without risks and quality of life sequelae [21]. Unfortunately, a majority of OPSCC patients present with locoregionally advanced (LA) disease for which a multimodality therapeutic approach including radiotherapy (RT) or chemo-RT (CRT) with or without surgery is usually employed [22,23,24]. 

CRT avoids surgery-associated risks, but there are frequently other complications including widespread tissue damage/toxicity in the head/throat region, including significant impacts for speech and swallowing, plus increased risk for secondary malignancies [25]. Furthermore, the use of cetuximab, a monoclonal antibody against epidermal growth factor receptor (EGFR), in combination with cisplatin or carboplatin and 5-fluorouracil (5FU) as a first-line treatment of recurrent/metastatic (R/M) HNSCC (EXTREME) has some utility with improved overall survival compared to platinum plus 5FU treatment with a response rate in about one third of patients [26]. Clearly there is a requirement to minimise toxicities and avoid the late complications, particularly in the younger patients with HPV-positive OPSCC, with its better prognosis [7,27]. To summarize, OPSCC generally receive RT or surgery for early-stage tumour treatment with combination therapies used for more advanced cancers.

Consequently, in spite of improvements in diagnostics, treatment, and surveillance, the 5-year progression-free survival (PFS) of patients with HPV-negative OPSCC with LA disease is ~40–50%, and survival rates for R/M disease have not significantly changed over the past years [5]. Advanced-stage HPV-positive OPSCC patients have five-year survival rates of 75–80%, vs. <50% among patients with similarly staged HPV-negative tumours [7]. With R/M disease having such a poor prognosis there is clearly an urgent need to identify biomarkers for detection of early disease, accurate prediction of prognosis, and appropriate selection of therapy. 

In 2018, The National Comprehensive Cancer Network Clinical Practice Guidelines in Oncology (NCCN, USA) defined some modifications of treatment plans for p16-positive vs. HPV-negative OPSCCs [28]. However, the options recommended in most cases did not vary except where as an alternative to definitive RT alone or surgery alone, treatment with RT plus systemic therapy was recommended for T1 N1 p16-negative but not for p16-positive tumours with a single node ≤ 3 cm. Overall, all OPSCC should receive RT or surgery for early-stage tumour treatment with combination therapies used for more advanced cancers. For example, platinum-based chemotherapy (CT) with RT reduces HNSCC and the OPSCC subgroup patient mortality by 26%, yielding an 8% absolute 5-year survival benefit [29]. However, using cisplatin plus RT showed significant increases in both acute and late toxicity. There was a lot of enthusiasm to test whether a de-escalation of SOC therapies could deliver a significant reduction in the side effects (toxicity) of the treatments plus an improvement in quality of life, but without any loss of efficacy. The logic was that the distinct tumour biology of HPV-positive OPSCC with their better prognosis derived from a possibly higher treatment sensitivity.

Several early phase clinical trials investigated whether response to induction CT could select patients with stage III-IV HPV-positive OPSCC for reduced-dose radiation. Indeed, patients with a complete response to induction CT retained cancer control with reduced radiation doses, but with reduced swallowing and nutritional problems [30,31]. Additional trials are probing de-escalated treatments for HPV-positive OPSCC. These include reduced-dose radiation and/or CT, stratifying by response to induction CT, stratification for any follow-up loco-regional therapy, efficacy of CT or RT as alternatives to surgery, minimally invasive transoral robotic surgery using pathology to stratify patients for de-escalation (and using targeted therapies) [28]. One such randomised, prospective clinical trial recruited only patients with LA HPV-positive OPSCC and was designed to investigate non-inferiority of efficacy accompanied by reduction in acute and late therapy toxicity where cetuximab was substituted for cisplatin [32]. Previous work had provided support for the value of cetuximab when added to RT for locoregional control and survival in LA HNSCC [30]. Unfortunately, the RT plus cetuximab treatment was inferior to SOC (RT, cisplatin) and increased the risk of cancer progression and death and loss of locoregional control without any significant influence on overall toxicities [32]. It is worth pointing out, however, that this needs to be tested in HPV-negative OPSCC where EGFR amplification, overexpression, and downstream signalling are more frequent in contrast to HPV-positive cancers, where mutations downstream of EGFR (e.g., activating in PIK3CA) are more frequent and could mediate resistance to cetuximab.

The recognition of the potential of immunotherapies in cancer treatment, including checkpoint inhibitors, has stimulated the investigation of biomarkers relating to immune parameters and their importance in HNSCC. The next sections discuss the evidence of immune dysfunction in OPSCC and the use of checkpoint inhibitors in treatment. The value of immune biomarkers assessments in the optimal deployment of treatments involving checkpoint inhibitors for OPSCC treatment, especially due to the differences in outcome depending on HPV status, is also discussed. 

## 4. Biomarkers in OPSCC

Investigation and validation of any potential tumour biomarkers could be of great value in prognostication and therapy selection and refinement for patients with OPSCC. There are challenges in utilising the extensive genomic information for biomarker application which arise from heterogeneity in the patterns of expression both within individual tumours and between HNSCC tumours. Differences in clinical outcomes for OPSCC are likely to involve multiple factors including differential radio/chemosensitivity and genetic heterogeneity [33,34,35], but it is also possible that activation of local oropharyngeal immunity has a role in limiting the spread of the disease and/or enhancing response to therapy. 

The important role of the immune system in the surveillance and control of neoplasia is now widely recognized [36,37]. Additionally, the subversion of such immune control through acquisition of immune suppressive factors in the tumour microenvironment (TME) is established as significant to cancer progression and poor treatment response, but understanding this also offers therapeutic opportunities [38,39,40,41]. The complex interactions at play in the natural history of an OPSCC are reflected in the emerging gene-expression profiles, which distinguish epithelial-mesenchymal transition (EMT), immune-related, keratinocyte differentiation, and oxidation stress factors with possible links to differential outcomes under investigation [4]. Detailed investigations of the types and proportions of immune cells and their interactions in the TME and any links to clinical performance following different treatments could provide the means for improved patient management. Any practical biomarker must have sufficient specificity and sensitivity, allowing clinically useful positive and/or negative predictive value, and be implementable in the clinical setting. 

## 5. Immune Deviation

### Lessons from Cervical Neoplasia

The lack of identifiable or accessible dysplastic precursor lesions limits our understanding of the natural history of HPV-driven neoplasia in OPSCC. However, based on our knowledge of HPV-associated cancers of the anogenital tract, in particular cervical neoplasia, there is clear evidence that the hr HPV infection of the target epithelium and the viral oncogene expression undermines the functioning of key elements of the innate immune system. This begins with a reduction of antigen-presenting cells which allows early virus immune evasion and persistence of infection. Over time, a multitude of interacting and self-reinforcing events actioned through modulation of different immune receptors, chemokine, and cytokine responses combine to generate an immune-suppressive microenvironment. This includes infiltration of immune-suppressive populations of Tregs, Th17, myeloid-derived suppressor cells (MDSCs), and upregulation of immune checkpoint receptor/ligand pathways [40,41,42]. All these changes constitute an immune deviation which can critically undermine the key T cell effector mechanisms capable of tumour control and elimination.

## 6. Immune Infiltration of OPSCC

### 6.1. T-Lymphocytes 

Many investigations of whether the densities of various TME-infiltrating immune cells correlate with clinical outcomes in HNSCC have been performed [43,44,45,46]. Unsurprisingly, the inherent heterogeneity of the HNSCC with different prognoses plus the diversity and quality of immunohistochemical (IHC) analyses performed have yielded a wide range of results. A priori, the acquisition of diverse and cumulatively immunosuppressive factors will impact on tumour control and clinical performance in therapy. The question here is whether there are biomarkers from this set of parameters that can be useful for patient management, etc. Here, the focus is on OPSCC, where there are clear differences in patient clinical outcomes for HPV-positive and HPV-negative tumours. 

Overall, there is a consensus for high levels of CD8+ and CD4+ T lymphocytes associating with better clinical responses to SOC treatment [46]. Thus, higher levels of infiltrating T cells have been documented in HPV-positive compared with HPV-negative OPSCC [47,48,49,50] (but not always [51,52]). However, when an increased T cell density was found in HPV-positive OPSCC it did not necessarily correlate with improved outcome. Interpretation of the results would be easier if there was consistency in the HPV typing methods and the techniques and reagents used to measure different immune factors in the TME. These should in particular include the precise location (tumour or stroma), the functionality of potential effectors, or the presence of other influences (secreted or cellular) which might counteract any anti-tumour activity. A study that assessed the relative infiltration in the tumour and the associated stroma reported a significant correlation of stromal infiltration by CD8+ T cells with better clinical outcome in HPV-positive OPSCC [43]. Evidence from cervical neoplasia has associated T cell infiltration of the epithelium as the key to lesion regression [53] and that the stroma may be the point of entry for such effector cells [54,55], possibly associated with activation of relevant gene expression in the stroma [56].

Of course, an alternative tumour-driven inflammatory influence can lead to the secretion of cytokines able to recruit other types of immunosuppressive cells to the TME. For example, Tregs contribute to control of immunological tolerance but can also play a role in facilitating cancer progression through suppression of anti-tumour immune responses. For HNSCC, including OPSCC, there is conflicting evidence regarding their role in suppressing tumour immunity and survival [57]. In part, this reflects the inadequacy of the markers used to identify Tregs either in the tissues or the peripheral blood plus lack of information on their functional status [46]. 

The inflammatory milieu of the TME plus the relative proportions of, e.g., T cell effector or suppressor cells populations are likely to influence the clinical outcomes of the patients. For example, studies have associated higher interferon gamma but lower interleukin-4 and transforming growth factor-beta levels in HPV-positive compared to HPV-negative OPSCC [58]. The presence in the TME of IFN-gamma-producing CD4+ and CD8+ T cells, inferred from expression of the transcription factor Tbet, has shown association with improved OS [45,59], while the presence of non-T cell IL-17-producing cells was linked to poorer survival [60]. Paradoxically, in one study, Tbet-positive Tregs were shown to accumulate in HPV16-positive OPSCC and the patients showed improved survival to SOC therapy [61]. Importantly, in such good responder patients, the Tbet-expressing intratumour populations of Tregs correlated with the levels of Tbet-positive CD4+ and CD8+ T cells and the detection of HPV-specific T cells. Such results are consistent with the characteristics of a T helper 1 type biased immune response with balance of inflammatory conditions supporting anti-tumour activity [61]. 

The functionality of anti-tumour effector T cells in the TME can also be compromised by alterations in HLA expression by the tumour target cells [62]. In HPV-driven cancers, overexpression of E6/E7 oncogenes facilitates genomic instability, and any generated mutations that cause HLA class I down-regulation by tumour cells could be selected as a means for immune escape. However, such variation in HLA class I expression in HNSCC does not appear to correlate with clinical outcome [63,64]. Once again, the individual influence of a gross measure like HLA class 1 expression may be compromised by the heterogeneity and the integrated effect of a multiplicity of contributing factors. Interestingly, upregulation of HLA class II expression on HPV-positive OPSCC has been correlated with improved survival of such patients, consistent with a role for CD4 T cells in the control of OPSCC [65].

### 6.2. Myeloid Cells 

Notably, the particular flavour of the inflammation can also influence the myeloid cell infiltrate and its functionality in the TME including by differentiation into macrophages, myeloid-derived suppressor cells (MDSC), or antigen-presenting dendritic cells (DC). Thus, when blood monocytes attracted to the TME differentiate into tumour-associated macrophages (TAM) they can provide either anti-tumour (M1) or tumour-promoting activity (M2) as driven respectively by the local levels of IFN gamma versus IL-4/IL-13 [66]. Furthermore, various secretions (TGF-beta, vascular endothelial growth factor (VEGF), IL-6, IL-10, prostaglandin E2 (PGE2), and chemokines) by the M2 macrophages act to promote tumour metastasis and Treg expansion with concomitant suppression of effector T cell activity [67]. Most importantly, in response to local IFN gamma production or hypoxia, TAM can upregulate inhibitory ligands of the immune checkpoint pathways which can limit anti-tumour T cell activity (see below). It is no surprise that there are investigations of OPSCC macrophage tumour infiltration and clinical outcome which support either a role in tumour control [67] or in promotion [65]; this can also differ in HPV-negative vs. HPV-positive tumours [63]. This lack of clarity most likely derives from the difficulty of distinguishing the functionality of macrophages in the context of all the other contributing factors. Some commonality for a positive contribution by TAM is probably favoured by the Th1 cell response flavour of local immunity in the TME [67]. 

MDSCs can be recruited from the blood or generated locally by arresting monocyte differentiation at an immature point. They can accumulate in different places in response to proinflammatory molecules (PGE2, IL-1β, IL-6, VEGF, S100A8/A9 proteins, and the complement component C5a) produced by tumour cells or by host cells in the TME [68]. MDSCs suppress anti-tumour immunity through a variety of diverse mechanisms. T cell activation is suppressed by production of arginase and ROS, the nitration of the T cell receptor (TCR), cysteine deprivation, and the induction of Tregs. Innate immunity is impaired by the down-regulation of macrophage-produced IL-12, the increase in MDSC production of IL-10, and the suppression of natural killer (NK) cell cytotoxicity. Antigen presentation is limited by the expansion of MDSC at the expense of DC. Defining the monocytic MDSC phenotype requires multiple markers (CD14+ HLA-DR-CD33+ CD11b+) that are difficult to assess in TME in situ but can be assessed by flow cytometry to enumerate circulating levels [69,70]. High systemic levels of MDSC have been associated with recurrence in OPSCC patients [71], and a variety of depletion strategies are being investigated to potentially boost immunotherapeutic strategies [72]. A recent study has highlighted the need to monitor the dynamics of monocyte differentiation in the context of other populations of peripheral blood mononuclear cells (PBMC) [73]. Thus, an elevated monocyte count was detected before the initiation of treatment in the PBMC of OPSCC patients which correlated with poor prognosis. Dynamic changes in monocyte subsets were documented involving the immune checkpoint ligand PD-L1 expression on monocytes and monocyte-derived MDSCs. Other studies have investigated peripheral blood measures of T effector lymphocytes, but this has not generally correlated with the levels in the TME or reliably predicted clinical outcomes [51].

Finally, monocytes recruited into the TME can also differentiate into DCs and high numbers can be associated with good clinical outcomes [74]. As the primary antigen-presenting cells in the tumour, they can promote the numbers and sustained functionality of infiltrating cytotoxic T lymphocyte responses. It is generally thought that DCs in tumours capture tumour antigens and migrate to draining lymph nodes, where they prime and activate tumour-specific T cells. Memory and effector CTLs return to the tumour to perform immunosurveillance activities limiting tumour development and/or progression. Infectious disease models have established that antigen-experienced T cells require cognate interactions with tissue DCs to expand in situ and achieve full effector functions. Unfortunately, due to the immunosuppressive nature of the TME, plus their plasticity, tumour DCs are often dysfunctional, which in turn facilitates immune evasion. Indeed, it appears that the number, phenotype, and function of DCs can alter as a cancer develops with tumour-infiltrating DCs, macrophages, MDSCs, and T cells densities increasing but with a change from an immunostimulatory to an immunosuppressive DC phenotype [75]. 

## 7. Immune Checkpoint Pathways 

Multiple inhibitory (e.g., PD-L1, TIM-3, LAG-3, CD200, and CTLA4) and activating (4-1BBL, ICOS-L, CD80, and CD86) immune regulatory molecules influence the activation, differentiation, and proliferation of T cells. T cell interactions with immature DCs can lead to T cell tolerance through various mechanisms, including deletion, anergy, and the generation of regulatory T cells [76,77]. Importantly, a class of inhibitory agents targeting some of these immune checkpoint pathways has been associated with sustained tumour responses in a variety of cancers [78]. The receptor-programmed death−1 (PD-1) interacting with its ligand PD-L1 is recognized as one dominant negative regulator axis of anti-tumour effector function whereby the interaction of PD-L1 leads to PD-1-mediated T cell exhaustion and inhibition of anti-tumour cytotoxic T cells. The stimulation of tumour specific T cells in the local tumour environment releases interferon gamma and this upregulates PD-L1 on the local tumour and other cells. This type of homeostatic mechanism is usually deployed to limit inflammation in a resolving lesion, but in the chronic tumour situation it can be subverted to compromise T cell function through adaptive immune resistance as discussed in more detail by others [79]. For more information on the subject, please also see a recent excellent review on emerging concepts in PD-1 checkpoint biology [80]. 

Blockade of the PD-1/PD-L1 axis by antibodies can reverse this immune suppression, and such single-agent therapies are now licensed treatment for several types of cancers [75,81]. Expression of PD-L1 in tonsillar crypts has led to speculation that it might facilitate HPV infection at these sites and has therefore encouraged the use of PD-1 inhibitors in patients with HPV-positive tumours [82]. Some studies have shown that upregulation of PD-1 and/or PD-L1 in OPSCC is linked to stronger immune infiltration and a good prognosis following SOC treatment, possibly reflecting an effective ongoing anti-tumour immune response [83,84,85,86]. However, a recent meta-analysis of immunohistochemistry (IHC) analyses for PD-L1 expression showed a positivity rate of 36–48% for HNSCC; but while HPV-positive tumours showed a relatively increased PD-L1 expression, in neither case was the PD-L1 level linked with OS [87].

## 8. Licencing of Immune Checkpoint Inhibitors for Treatment in HNSCC 

Building on encouraging early phase clinical studies in HNSCC, late phase III trials have now executed validation studies of several immune checkpoint inhibitor antibodies that block the PD-1/PD-L1 axis for the treatment of R/M HNSCC patients that failed a platinum-based therapy [88]. The illustrative results are summarized in Table 2 for two PD-1 inhibitors, both humanized IgG4s, nivolumab (OPDIVO, Bristol-Myers Squibb, New York, NY, USA), and pembrolizumab (KEYTRUDA, Merck Sharp & Dohme, Kenilworth, NJ, USA), plus durvalumab (high affinity engineered IgG1 versus PD-L1). The phase III studies for nivolumab (CHECKMATE-141) [89] and pembrolizumab (KEYNOTE 040) [90] both showed a survival benefit as compared to standard monotherapy for diseases progressing less than 6 months after a platinum-based CT [88,89,90]. Long term results of the nivolumab CHECKMATE-141 study (minimum follow-up 24.2 months) confirmed a survival advantage with the 2-year rate almost 3-fold more, an increased durability of response, and much less toxicity [91]. For the KEYNOTE 040 study, the primary endpoint of increased OS was met with a median of 8.4 months for the pembrolizumab arm compared to 6.9 months in the standard arm, respectively, and first-year survival comparison was 37% vs. 26.5%. The response rate is moderate at 14.6% in the pembrolizumab arm vs. 10.1% in the SOC arm, but with a very long median response duration in the pembrolizumab arm of 18.4 months and a better toxicity profile. 

It is important to point out that any direct comparison of these trials is complicated by subtle differences in the patients recruited and the treatments provided in the standard therapy arms. However, the efficacy of these drugs in patients with R/M HNSCC revealed a relatively low overall response rate with no difference in response between HPV-positive and HPV-negative cancers. By apparent contrast, durvalumab in the EAGLE phase III study showed no significantly increased OS compared to SOC treatment, but this negative result probably results at least in part from unexpectedly good outcome in the control arm (SOC) patients. Interestingly, there is a lot of mortality in the early stages of this trial in the immunotherapy arm patients, but the fraction of these patients who survived showed increased duration of responses, better survival at 2 years, plus reduced toxicity to durvalumab relative to the SOC arm [92]. 

Unfortunately, the impact of immune checkpoint inhibitors is somewhat limited by the relatively low proportion of patients who respond, the need to manage potential autoimmune toxicities in some patients, as well as the high treatment costs. This highlights the value of a biomarker(s) that might allow for selection of patients who could most benefit from such immunotherapies. The obvious candidate has been to investigate whether the level of inhibitory molecule tumour expression can predict patient responses.

## 9. PD-L1 Expression as a Prognostic in HNSCC and in Association to HPV Status

PD-L1 expression has been widely investigated for use as a predictive marker in several solid tumours although the results have had limited value in clinical application thus far [93]. This is seen with HNSCC, where investigations of PD-L1 positivity within a tumour have not yielded consistent results in seeking any correlation to clinical responses. This reflects the problems derivative from the heterogeneity of tumours included in different studies (HNSCC, OPSCC, HPV status), the associated patient treatment schedules, as well as the reagents and detection methodologies, the definitions of positivity, the cut-off levels used, and the scoring system with inclusion of tumour cells alone (TPS: tumour proportion score) or all relevant cells expressing PD-L1 (CPS: combined proportion score). In addition, our current understanding of the biology of immune checkpoint control and the mechanisms of the agents that block these pathways is still very limited.

Even investigating the more homogeneous OPSCC tumours and stratifying by HPV status has been challenging for integration and interpretation of simple density measures of different cell types showing PD-L1 expression in TME with clinical outcomes to SOC. For example, populations identified as CD8+ T cells and CD68+ macrophages were both higher in HPV-positive compared to HPV-negative OPSCC. However, when subpopulations were identified with additional markers of the checkpoint pathway CD8+/PD-1 T cells were at similar levels but the proportion of CD68+/PD-L1/macrophages was lower in HPV-positive compared to HPV-negative OPSCC [94]. Indeed, in HPV-negative OPSCC patients, the increased PD-L1/macrophage levels were associated with better outcome [94].

Instead of simple enumeration of cell densities, analysing the spatial organisation of cells in the TME may be able to provide a more “functional” measure of immune regulation. Thus, an automated analysis pipeline was used to quantify the potential of T cells to interact with PD-L1 expressing cells in the TME. An algorithm was used that discarded artefacts and scanning errors, performed cell segmentation, and determined events defined by the proximity between cell subsets employing the hypothesised interaction distribution method. The results showed that a high frequency of spatial interactions between CD8+- or PD-1-marked T cells and PD-L1^-^positive cells were prognostic for poor overall survival in patients with HPV-negative OPSCC [95]. Previous analyses of the same cohort using density measures of PD-L1 expression with CPS >5% had concluded that only stromal PD-L1 was prognostic in HPV-negative OPSCC [94]. However, none of these results support the notion that PD-L1 expression levels link to improved outcome in a consistent way. 

## 10. PD-L1 Expression to Stratify Treatment 

In the context of immune checkpoint inhibitor trials in HNSCC, IHC-assessed PD-L1 expression was scored on either tumour cells alone (TPS) or also including tumour-infiltrating immune and stromal cells (CPS). For example, in CHECKMATE-141 [89,91], a PD-L1 tumour membrane expression of ≥1% expression was the positive definition using the Dako PD-L1 IHC 28–8 pharm Dx test. This concluded that there was a greater positive impact on outcome for nivolumab-treated patients compared to those receiving standard therapy if their tumours expressed PD-L1 and compared to those with PD-L1-negative tumours. However, by 2 years of follow-up, checkpoint inhibitor treatment also showed evidence of benefit in the patients with PD-L1-“negative” tumours. Refining the definition of positivity for the PD-L1 expression scores of ≥1% vs. ≥5% vs. ≥10% showed increased overall response rates, but did not alter the OS measures [89]. Clearly, this biomarker methodology cannot identify all the patients that could respond to treatment. When the presence of PD-L1-expressing tumour-associated immune cells were included in the scoring, this was shown to be more predictive of benefit than tumour cell PD-L1 expression alone, but this was complicated by the observation that the predicted increased benefit from nivolumab compared to SOC was greater in tumour cell PD-L1-negative patients [91]. For KEYNOTE-040, PD-L1 tumour expression was assessed by a different test (Dako PD-L1 IHC 22C3 pharmDx). In exploratory analyses, not adjusted for multiplicity, an interaction between the treatment effect for OS and PD-L1 expression was seen, with the benefit of pembrolizumab greater in patients with a CPS ≥ 1 compared to CPS < 1; this was also seen when stratifying tumours by TPS ≥ 50% versus < 50%. These preliminary results suggest benefits on PFS and objective response of checkpoint inhibitor versus SOC therapy which are greater in patients whose tumours had higher PD-L1 expression [90]. Further studies assessing PD-L1 positivity (CPS ≥ 1) showed a significant improvement in OS compared to SOC [3,96]. These phase III studies in patients with R/M HNSCC for nivolumab (CHECKMATE-141) and pembrolizumab (KEYNOTE-040) compared to SOC led to FDA/EMA approval; the EMA subsequently restricted pembrolizumab to patients with tumours scoring TPS ≥ 50% [3]. 

In a further development, the KEYNOTE-048 study investigated the use of pembrolizumab as first-line treatment for platinum-sensitive patients with R/M disease. This showed that compared to SOC, CT (platinum + 5-FU), pembrolizumab, plus CT provided a better OS in the PD-L1 CPS ≥ 20, CPS ≥ 1, and total populations, while pembrolizumab monotherapy demonstrated superior OS in the CPS ≥ 20 and ≥ 1 populations and was non-inferior in the total population [3,97]. The FDA has approved pembrolizumab for CPS ≥ 1 patients and for pembrolizumab and CT for the entire population, but the EMA restricted its use to CPS ≥ 1 patients receiving pembrolizumab alone or with CT. The 2-year survival rates of 29–35% are significantly improved compared to the EXTREME regimen, notwithstanding the reduced toxicity. Response rates are still less than 23% with 40.5% progression for pembrolizumab alone, so there is still plenty to do to increase efficacy and focus such immunotherapy. Importantly, the response rate to pembrolizumab plus CT of 35% does not improve on that delivered by CT alone while the nearly doubled survival rate of 30%–35% is about the same as the immunotherapy alone [97].

Here, only some of the pivotal clinical trials have been described to highlight the issues of utilising biomarkers in immune checkpoint inhibitor treatment selection. There are more details of the evolution of these, and many other supporting studies in Borel et al. 2020 [88]. While there is a general correlation of PD-L1 tumour expression with immune checkpoint inhibitor efficacy in R/M HNSCC, with improved predictive quality when using CPS, it is still clear that some patients whose tumours area scored as PD-L1-negative can still benefit from treatment. Comparative studies of reagents and methodologies are being conducted, but so far without any definitive conclusions. 

## 11. General Reflections on Biomarkers for OPSCC

It does appear that right now there is a limited value in IHC-based assessments of PD-L1 expression for accurately determining treatment options. The challenge is how to interpret measures (e.g., density) of different populations of immune and other cell types and their functional status from 2D snapshots accounting proximity and the presence of soluble factors that will be integrated to contribute to “responsiveness”. Ultimately, multiplex IHC techniques will have a limit of simultaneously analysable markers even when utilising automated scanning and scoring systems. How likely is any combination of factors measured by such mainly IHC studies ever going to reflect the nuances of the multitude of components that are dynamically integrating to determine potential responsiveness to a checkpoint inhibitor in an OPSCC patient? Given the number of other known regulatory pathways active in the TME and systemically, plus undoubtedly unknown unknowns, the failure to identify a really specific measure of PD-L1 expression for very accurate prognostics and treatment deployment in HNSCC (OPSCC) is probably understandable. 

In other words, current biomarkers like those being investigated above do not provide a sufficiently granular distinction between one tumour and another to achieve effective stratification of treatment. It follows that until studies can be conducted with more “granular” eligibility (biomarker profile) for checkpoint therapy, it will be difficult to identify cohorts who can best benefit from this approach.

The analyses for PD-L1 so far have been based on a reasonable hypothesis whereby more PD-L1 expression in the tumour reflects a history of undermined immune control which is available to be released on checkpoint inhibition. Some have hypothesised and found general evidence supporting the idea that there is a correlation between the number of accumulated mutations, thereby generating potential tumour antigens, and the response to checkpoint inhibitors [98,99,100,101]. T cell infiltration in tumours is also well correlated with improved outcomes with Immunoscore providing useful prognostic information in colorectal cancer [36]. 

Future progress in determining better prognostic and therapy application tools may require the integration of additional classification features of a tumour incorporating the mutational load, (epi) genetic background (microsatellite instability) status, chromosomal instability, and CpG island methylator phenotype status, as well as risk factors like virus or other infection, UV, chemical, and physical carcinogen exposure [102]. Such investigations emphasize the importance of the immune context but also the extent of the positive and negative factors shaping carcinogenesis and tumour progression or control. A recent study has focused on the balance of PD-1 positive CD8+ T and Treg cells in cancers and shown that the ratio in the TME can predict the clinical efficacy of PD-1 blockade therapies compared to PD-L1 expression or mutational burden [103]. It is hypothesized that a better recovery of dysfunctional PD-1-positive CD8+ rather than Tregs is required for tumour regression. This once again highlights the relative lack of predictive power with such approaches. 

The functionality of infiltrating T cells effectors induced by chronic stimulation, typically viral, can also lead to a state of exhaustion [104]. Such T cells over-express several inhibitory receptors (e.g., PD-1), exhibit major changes in T cell receptor and cytokine signalling pathways, have changes in expression of genes controlling chemotaxis, adhesion, and migration, and show distinct transcriptional signatures and various metabolic and bioenergetic deficiencies [105,106]. The continuous stimulation of naïve T cells in the local lymph node leads to a skewed population of CD8+ effectors at lesion sites with functional T cell exhaustion progressive but distinct from anergy [107]. A homeostatic mechanism is invoked that aims to protect the repertoire of antigen-specific T cells by generating a stem cell-like CD8+ non-recirculating population which resides in T cell zones of lymphoid tissues along with the naïve T cells [108]. They are quiescent but have proliferative potential and provide a means to sustain supply of activated specific T cells in a chronic situation. Tcf-1 is critical for generation of this PD-1-positive CD8+ T cell subset which can permit self-renewal as well as differentiate into more terminally differentiated cells that down-regulate Tcf-1 and with a transitory population of CD101-negative Tim3-positive cells can convert to CD101-positive Tim-positive T effectors. Importantly, PD-1 pathway blockade increases the numbers of transitory cells, suggesting that these cells play a critical role in PD-1-based immunotherapy [106]. While these novel sets of both terminally differentiated and stem-like CD8+ T cells have mostly been investigated in chronic virus infection they have also been detected in human tumours. Importantly, they are found in pseudo-antigen-presenting-cell niches within some tumours and, interestingly, patients with progressive disease seem to lack these immune niches and this correlates with low levels of T cell infiltration [109,110]. It appears that the mechanisms underlying checkpoint blockade depend not on a simple reversal of T cell exhaustion but requires the expansion of the stem-like population [110,111,112]. When these cells are released from their quiescence, they are subsequently receptive to antigen presentation, and can mobilize, proliferate, and functionally differentiate. The checkpoint blockade may also influence effector function at the target sites with increased killing and cytokine release. A greater understanding of how to best mobilize this unique stem-like population in a chronic viral-driven tumour will be important to maximizing the impact of therapies which seek to harness the immune response [41].

Finally, using dissociated OPSCC tissues and more powerful techniques based on mass spectrometry and/or single-cell RNA analyses may provide for the use of more co-markers to improve precision in identifying particular populations like CD103+ tissue-resident T cells whose presence and function may be pivotal in anti-tumour immunity [74,112,113,114,115]. However, the analyses may be compromised by the process of recovery altering the cellular RNA, etc. expression status. Nevertheless, such approaches could be useful in revealing important features of the TME critical to outcomes in response to available treatments. There are also platforms available which can integrate multiplexed expression data (RNA, protein) from particular individual cells in the context of the tissue with the option to look for dynamic changes seen in disease progression or treatment [116,117,118]. 

On the other hand, the practicality of these more sophisticated techniques to routine assessment for either prognostics or treatment allocation is more doubtful through logistic and cost issues, at least in the short term. This article has therefore concentrated on immune factors assessed locally in the context of checkpoint inhibitors as a central pivot in current therapeutic treatments in OPSCC. More global analyses of gene expression patterns have been applied to define different HNSCC subtypes incorporating biological characteristics and de-regulated signalling pathways as immunoreactive, inflammatory, HPV-like, classical, hypoxia-associated, and mesenchymal with the latter two more aggressive [103]. More information does not necessarily provide useful clinical insights if the subgroups are too numerous and still heterogeneous [119,120].

## 12. Types of Combination HNSCC (OPSCC) Therapies under Investigation 

In the context of checkpoint inhibitor treatments for HNSCC (OPSCC) there is established proof of principle for improved management which warrants continued research for useful biomarkers enabling better prognostics and treatment deployment. To a large extent, biomarker studies are mostly an add on, or evaluated post hoc, in clinical trials primarily investigating the efficacy of increasing numbers and varieties of immune checkpoint inhibitors delivered in combination with variations of SOC or with other experimental immuno-therapeutic strategies. It is sobering to consider that a fundamental re-evaluation of the influence of radiation and chemical treatment protocols on both local and systemic functional immunity should really be reconsidered in the context of the deployment of immunotherapies aimed at either recovery of an existing immune response or the induction of novel anti-tumour activity [121,122,123]. The ongoing clinical studies of checkpoint inhibitors in HNSCC have recently been reviewed by the van der Burg group [46] and can be comprehensively accessed at Clinicaltrials.gov [124]. The extent and diversity of approaches reveals a somewhat scatter-gun approach which may luck out but could also just consume resources for a minimal gain. A summary of a selection of ongoing later phase 2/3 trials outlining the design, size, patient eligibility criteria, primary endpoints, and their timelines is given Table 3. These relatively few examples illustrate the challenges of designing meaningful clinical studies to investigate PD-1/PD-L1 pathway inhibition in the context of suboptimal SOC with a view to improved patient management/outcomes. Is it the case that this activity is only likely to further progress incrementally for highly defined groups and that for the majority of patients real progress will require new treatment paradigms based on new knowledge? 

## 13. Final Reflections

Many drugs targeting costimulatory and coinhibitory immune checkpoint molecules have now been developed. How conventional therapy influences these pathways in different immune cell subsets and beyond during the course of treatment is largely unknown. Some recent studies have begun to investigate such effects and may help to identify possible novel combinational therapeutic approaches [125,126]. The caveat is that these regulatory networks are often interactive (cellular and extracellular) and provide a redundancy that allows for fine tuning in responses, so insight from only a few biomarker assessments may be relatively limited. Combination treatments based on understanding the influence of immune deviation on tumour survival have utilised initial chemotherapy to provide for a reduction in the levels of MDSCC followed by HPV 16 oncogene vaccination which is then more effective at stimulating anti-tumour T cell responses, enabling prolonged survival [127,128]. Likewise, checkpoint blockade in combination with tumor-specific vaccination of patients with HPV16-related cancer has been attempted and has shown some promise [129]. Going forward, especially by utilising technology advances, additional modification strategies will be investigated to improve targeting and stimulation of cytotoxic T cell-based immune responses for optimal development of combination therapies [130,131].

A useful biomarker must identify a reasonable proportion of the patients where assignment of the associated treatment option has a high certainty of clinical value and where others are not excluded from this potential benefit. Ultimately, protocols which provide the best options for maximising response and minimising toxicity for the largest number of patients to be effectively treated are empirically determined in iterative clinical studies. The more factors used to refine the options for therapy selection, the more stratification of the patients occurs, ultimately leading to individually personalized medicine which must drive up costs even if logistically deliverable. Hopefully, the extensive testing of combinations of therapeutic approaches by types and sequencing will fuel the next evolution of treatment for OPSCC (HNSCC), albeit through the stalwarts of much medical progress, empiricism, and serendipity. 

## Figures and Tables

**Table 1:** AJCC prognostic clinical or pathological stage groups for HPV-related (p16-positive) oropharyngeal cancers [10,11].

Stage	Clinical	Pathological
I	T0N1M0, T1N0M0, T1N1M0, T2N0M0 or T2N1M0	T0N1M0, T1N0M0, T1N1M0, T2N0M0 or T2N1M0
II	T0N2M0, T1N2M0, T2N2M0, T3N0M0, T3N1M0 or T3N2M0	T0N2M0, T1N2M0, T2N2M0, T3N0M0, T3N1M0, T4N0M0 or T4N1M0
III	T0N3M0, T1N3M0, T2N3M0, T3N3M0, T4N0M0, T4N1M0, T4N2M0 or T4N3M0	T3N2M0, or T4N2M0 IV Any T, any N and M1
IV	Any T, any N and M1	

Legend: A general categorization assigns cancers as stage 0 (abnormal cells are present but with no spread); stages I-III (increasing size and local spread), and stage IV (spread to distant sites). Additional information is provided by the TNM system: T refers to the size and extent of the main or primary tumour, N to the number of nearby lymph nodes that have cancer, and M to whether the cancer has metastasized. Further information is reflected in numbered subdivisions. Thus TX: main tumour cannot be measured; T0: primary tumour cannot be found; and T1, T2, T3, T4: refer to the size and/or extent of the main tumour. The higher the number after the T, the larger the tumour or the more it has grown into nearby tissues. Tumours may be further divided to provide more detail, such as T3a and T3b. Likewise, for regional lymph nodes (N): NX: cancer in nearby lymph nodes cannot be measured; N0: there is no cancer in nearby lymph nodes; N1, N2, N3: refer to the number and location of lymph nodes that contain cancer. The higher the number after the N, the more lymph nodes that contain cancer. For distant metastasis (M): MX: metastasis cannot be measured; M0: cancer has not spread to other parts of the body; M1: cancer has spread to other parts of the body. Recent modifications in the T classification for some tumours include the depth of invasion (DOI) of the primary (increase category by 1 level for each 5 mm of depth) and new refinements on the N categorisation utilising pathological (histology) and/or clinical (imaging) to measure extra-nodal extension (ENE). Cut-offs define changes in staging level or a new sublevel. Pathology-driven measures can be assessed and validated retrospectively, but this is more difficult for clinically based approaches for there is no precedent and the imaging modalities have very different sensitivities and specificities. For example, a clinical estimation of depth of invasion may overestimate by 1–2 mm, and contribution to size of lesion through inflammation or ulceration cannot be distinguished.

**Table 2:** Key immune checkpoint inhibitor clinical efficacy trials for HNSCC.

Study [Ref] (No of Patients)	Drug v IC or SOC	OS: Median (mo) HR (*p* Value)	1 Year OS (%) 2 Year OS (%)	PFS: Median mo HR (*p* Value)	Overall Response Rate (%) Median Duration (mo)	% Toxicity Grade 3
CHECKMATE-141 [84,86] (*n* = 506) Open Label, Randomized Phase 3 Trial of Nivolumab vs. Therapy of IC in R/M platinum-refractory HNSCC (NCT02105636)	Nivolumab v IC	7.5 v 5.1 0.70 * (*p* = 0.01)	36.0 v 16.6 16.9 v 6.0	2.0 v 2.3 0.89 (*p* = 0.32)	13.3 v 5.8 9.7 v 4.0	13.1 v 35.1
KEYNOTE-040 [85] (*n* = 495) Phase III Randomized Trial of Pembrolizumab vs. SOC in R/M HNSCC patients (NCT02252042)	Pembrolizumab v SOC	8.4 v 6.9 0.80 * (*p* = 0.0161)	37.0 v 26.5 NA	2.1 v 2.3 NA	14.6 v 10.6 18.4 v 5.0	13 v 36
EAGLE [87] (*n* = 736) Phase III Randomized, Open-Label, Durvalumab monotherapy (& combination with tremelimumab) vs. SOC in R/M HNSCC patients (NCT02369874)	Durvalumab v SOC	7.6 v 8.3 0.88 (*p* = 0.76)	37.0 v 30.5 18.4 v 10.3	2.1 v 3.7NA	17.9 v 17.3 12.9 v 3.7	10.1 v 24.2

Legend: NCT = National Clinical Trial; IC = Investigators Choice; HR = Hazard Ratio; mo = month; * statistically significant.

**Table 3:** Selected phase 2/3 clinical trials involving immune PD-1/PD-L1 pathway inhibitors in HNSCC(OPSCC).

NCT (ClinicalTrials.Gov)	Design	Treatment Arms	Patient Eligibility	Primary Endpoints	Start (Month/Year) Status
03082534	Open-label, non-randomized, multi-arm phase II trial of pembrolizumab combined with cetuximab for patients with R/M HNSCC	Treatment: Pembrolizumab/Cetuximab Cohort 1 (PD-1/PD-L1 inhibitor-naïve, cetuximab-naïve); Cohort 2 (PD-1/PD-L1 inhibitor-refractory, cetuximab-naïve); Cohort 3 (PD-1/PD-L1 inhibitor-refractory, cetuximab-refractory); Cohort 4 (cutaneous HNSCC)	83 HNSCC not amenable to curative intent therapy.	ORR at: 6 months Proportion of patients with partial or complete response in tumour burden	3/2017 Active
01810913	Randomized phase II/III trial of adjuvant RT with cisplatin, docetaxel-cetuximab, or cisplatin-Atezolizumab (anti-PD-L1) in HR HNSCC. First select the better docetaxel-based exptl arm to DFS over control arm 1. (Phase II) (COMPLETE 3/2020) To determine if combination of docetaxel-cetuximab & IMRT is superior for OS compared to standard cisplatin & IMRT in adjuvant treatment (Phase III) To determine if combination of atezolizumab, cisplatin, & IMRT is superior in terms of OS compared to standard cisplatin & IMRT in the adjuvant treatment HPV-negative HNSCC (Phase III)	Experimental: Arm 1 (IMRT, cisplatin) Experimental: Arm 2 (IMRT, docetaxel) Experimental: Arm 3 (IMRT, docetaxel, cetuximab) Experimental: Arm 4 (IMRT, cisplatin, atezolizumab)	613 HPV negative HNSCC	DFS (Phase II) up to 7 years OS (Phase III) up to 7 years	3/2013 Active
03174275	Multimodality therapy with induction carboplatin/nab-paclitaxel/durvalumab followed by surgical resection & risk-adapted adjuvant therapy for treatment of LA & surgically resectable HNSCC	Experimental: Low Risk Part 1: 6 weeks of induction carboplatin chemotherapy; Part 2: 2–6 weeks post-induction, tumour imaging, & surgical resection; Part 3: adjuvant durvalumab Experimental: Medium Risk Part 1: 6 weeks of induction carboplatin chemotherapy in combination with durvalumab; Part 2: 2–6 weeks post-induction, tumour imaging & surgical resection; Part 3: ipsilateral involved field radiation concurrent with cisplatin followed by durvalumab. Experimental: High Risk 6 weeks of induction carboplatin chemotherapy in combination with durvalumab; Part 2: 2–6 weeks post-induction, tumour imaging & surgical resection; Part 3 IMRT concurrent with cisplatin or SOC & then durvalumab	39 previously untreated, histologically proven, surgically resectable primary HNSCC stage III or IV (HPV+ or negative non-metastatic disease)	Pathologic CRR after induction chemotherapy with carboplatin, nab-paclitaxel, & durvalumab in previously untreated stage III/IV HNSCC amenable to surgical resection approximately 8-12 weeks after start of study treatment	6/2017 Active
03258554	Randomized phase II/III trial of radiotherapy with concurrent durvalumab vs. radiotherapy with concurrent cetuximab in LA HNCCC patients with contraindication to cisplatin	Active Comparator Arm: cetuximab, RT Experimental Arm: durvalumab, RT	474 LA HNSCC Not suitable for cisplatin treatment	DLT up to 4 weeks after RT PFS (Phase II) up to 3 years OS (Phase III) up to 3 years	8/2017 Active
03383094	Phase II randomized trial of radiotherapy with concurrent & adjuvant pembrolizumab versus concurrent chemotherapy in patients with advanced/intermediate-risk p16+ HNSCC	Active Comparator: Control-RT/cisplatin Experimental: RT/pembrolizumab	114 HNSCC HPV + (p16) high-intermediate risk disease	PFS up to 3 years	12/2017 Active
03410615	Non-comparative, randomized, phase II study of cisplatin plus radiotherapy or durvalumab plus radiotherapy followed by adjuvant durvalumab or durvalumab plus radiotherapy followed by adjuvant tremelimumab and durvalumab in LA HPV+ OPSCC	Active Comparator: Radiation/Cisplatin Experimental: Radiation/Durvalumab + Adjuvant Durvalumab Experimental: Radiation/Durvalumab + Adjuvant Durvalumab/Tremelimumab (Arm closed to accrual in 2019)	180 LA HPV + (p16) HNSCC	3 year event-free survival	1/2018 active
03468218	Studies effects of pembrolizumab & cabozantinib (protein kinase inhibitor) in treating R/M HNSCC.	Experimental: Treatment (pembrolizumab, cabozantinib)	53 HPV + HNSCC	ORR	3/2018 Active
03618134	Studies the side effects & how well stereotactic body radiation therapy & durvalumab (anti-PD-L1) with or without tremelimumab (anti-CTLA4) before surgery work in treating participants with HPV+ OPSCC	Experimental: Cohort I (SBRT, durvalumab, TORS, neck dissection) Experimental: Cohort II (SBRT, durvalumab, tremelimumab, TORS, neck dissection)	82 HPV+ (p16 IHC) OPSCC. T0-3 disease with gross disease amenable to R0 resection (TORS eligible); N0-N2b, disease confined to 2 cervical LN levels if adjacent.	Phase 1 safety-related adverse events up to 90 days PFS (Phase II from enrolment to the first occurrence of disease progression up to 2 years & AE incidence	8/2018 Active
03646461	Randomized, phase II testing efficacy of Ibrutinib (tyrosine kinase inhibitor) in combination with either nivolumab or Cetuximab (EGFR inhibitor) in R/M HNSCC	Arm A: Ibrutinib + Cetuximab Arm B: Ibrutinib + Nivolumab	39 R/M HNSCC not yet treated with EGFR inhibitors	Efficacy of Combined Therapies at 3 years	8/2018 Active
03669718	A blinded, placebo-controlled, randomized, phase 2 study in which subjects will be randomly assigned 1:1 to cemiplimab plus placebo or cemiplimab plus ISA101b.	Experimental: Active ISA101b & cemiplimab Placebo Comparator: Placebo and cemiplimab	194 R/M OPSCC HPV16 +, PD-L1+ (CPS ≥ 1). Patients suitable for first-line PD-1 blocking antibody & with disease progression on or after platinum containing chemotherapy.	ORR & treatment-related adverse events in 25 months	9/2018 Active
03799445	Studies side effects & best dose of ipilimumab (anti-CTLA4), nivolumab, radiation therapy in HPV+ OPSCC patients	Single Arm: Nivolumab, ipilimumab, IMRT	180 stage 1-II (p16+, HPV DNA or RNA+)	DLTs relating immunotherapy. For phase II: CRR (at 6 months & PFS at 2 years)	1/2019 Active
03829722	Does 2yr PFS improve with add of nivolumab compared to SOC fractionated RT & carboplatin/paclitaxel?	Single Arm: Nivolumab, Carboplatin/Paclitaxel, Radiotherapy	40 stage 3 (p16+) OPSCC	PFS up to 2 years	2/2019 Active
03952585	Does a reduced dose of radiation therapy & nivolumab (anti-PD-1) work as well as standard dose radiation therapy & cisplatin OPSCC patients?	Arm I: IMRT, IGRT, cisplatin Arm 2: Reduced IMRT, IGRT, cisplatin Arm 3: IMRT, IGRT, nivolumab	711 stage 1-II (p16+) OPSCC	PFS (Phase II/III) up to 6 years; QOL	5/2019 Active
03978689	Phase 1 dose escalation & expansion study evaluating the safety, anti-tumour effect, & immunogenicity of CUE-101 as monotherapy treatment in 2ndline or CUE-101 combination therapy with pembrolizumab in first-line HPV16+ R/M (HNSCC) patients. CUE-101 is a novel fusion protein designed to activate & expand a population of tumour-specific T cells to eradicate HPV-driven malignancies	Part A&B: First-in-human trial, to assess safety & tolerability of CUE-101 in subjects with R/M HNSCC in 2^nd^-line setting, to determine MTD or recommend Phase 2 dose based on markers of biological activity. PK, anti-tumour immune response, preliminary anti-tumour activity & immunogenicity will also be assessed. Part C&D: Characterize safety, tolerability, & biological effects of CUE-101 in combination with pembrolizumab in R/M HNSCC patients in first-line setting.	85 patients, HPV 16 + (RNA ISH & p16 IHC) R/M HNSCC progressed following at least 1 prior systemic therapy. HLA A * 0201 genotype	The primary objectives of the Part A&B, first-in-human trial, are to assess the safety and tolerability of CUE-101 in subjects with recurrent/metastatic HNSCC in the second-line setting and to determine the maximum tolerated dose or recommended Phase 2 dose based on markers of biological activity	6/2019 Active
04398524	Testing ISA101b (HPV 16 E6/E7 synethetic long peptide vaccine) plus cemiplimab in subjects who have progressed on prior anti-PD-1 therapy	Single arm: ISA101b 3 times plus cemiplimab every 3 weeks for up to 24 months	86 PD metastatic HPV16 + OPSCC at primary site & LNs limited to neck. Patients had at least 4 doses anti-PD-1 antibody with or without chemotherapy within 6 months.	Improvement in ORR after previous progression	5/2020 Active
04634825	Study of enoblituzumab (anti-B7-H3 targets B7 family immune regulatory molecule) combined with either retifanlimab (anti-PD-1) or tebotelimab (bispecific DART^®^ molecule designed to independently or coordinately block PD-1 & LAG-3 checkpoint molecules) given as first-line treatment to patients with R/M HNSCC	Arm 1: retifanlimab cohort (Enoblituzumab + retifanlimab) Arm 2: tebotelimab cohort Enoblituzumab + tebotelimab	80 R/M HNSCC 50 PD-L1+ve in retifanlimab cohort 30 PD-L1-ve in tebotelimabcohort.	Efficacy of enoblituzumab plus retifanlimab or enoblituzumab plus tebotelimab at 28 months Safety by 30 days after last dose. Incidence of treatment-emergent adverse events	11/2020 Active
04671667	Studies effect of pembrolizumab in combination with radiation therapy or pembrolizumab alone or SOC (chemotherapy plus radiation) in R/M HNSCC after surgery	Arm A: pembrolizumab, IMRT, PBRT Arm B: cisplatin, carboplatin, IMRT, PBRT Arm C: pembrolizumab	R/M HNSCC in a previously radiated field after surgery. HR disease with tumour PD-L1 (CPS) ≥ 1	OS at 2 years Adverse events up to 5 years	12/2020 Active
04718415	Studies efficacy & safety of sintilimab (anti-PD-1) in combination with carboplatin & nab-paclitaxel in patients with oral cavity or OPSCC who are about to undergo surgery.	Drug: sintilimab, paclitaxel, carboplatin Treatment repeats every 21 days for up to 2 courses in the absence of disease progression or unacceptable toxicity. Procedure: Surgical resection	OSCC or OPSCC which is planned for treatment with curative intent including surgical resection.	Adverse events up to 90 days Pathologic response to neoadjuvant treatment in resected tumour & lymph nodes compared to historical SOC tumours up to 6 weeks	12/2020 Active
04862650	Studies effect of cemiplimab (anti-PD-1) in combination with low-dose paclitaxel & carboplatin in R/M HNSCC	Single Arm: cemiplimab, paclitaxel, carboplatin)	33 R/M HNSCC	ORR at 12 weeks	NYA
04858269	Effects of carboplatin & paclitaxel plus pembrolizumab (anti-PD-1) in HNSCC patients unable to take 5FU	Single Arm: Pembrolizumab + carboplatin + paclitaxel in outpatient setting	35 R/M HNSCC not suitable for infusional 5FU	Do 6 cycles of pembrolizumab with weekly carboplatin/paclitaxel increase the radiographic response rate compared to historical rate for pembrolizumab alone?	NYA

Legend: [124] Abbreviations: RT, radiotherapy; IMRT, intense modulated RT; IGRT, image guided RT; DLT, dose limiting toxicity; CRR, complete response rate; ORR, overall response rate; PBRT, pencil beam RT; MTD, maximum tolerated dose; PL, pharmacokinetics; ISH, in situ hybridization; TORS, trans oral robotic surgery; DFS, disease control rate; NYA, not yet activated; OS, overall survival; PFS, progression-free survival; QOL, quality of life; IHC, immunohistochemistry. Drugs: PD-1 blocking antibodies: -nivolumab, pembrolizumab, cemipliamb, sintilimab; PD-L1 blocking antibodies: -retifanlimab, atezolizumab, durvalumab. Other immunotherapeutic agents: tebotelimab (bispecific DART^®^ molecule designed to independently or co-ordinately block PD-1 & LAG-3 checkpoint molecules; enoblituzumab (anti-B7-H3 targets B7 family immune regulatory molecule); anti-CTLA4 antibodies: ipilimimab, tremelimumab. Kinase inhibitors: ibrutinib, cabozantinib; Other immunotherapeutic agents: ISA101b, HPV 16 E6/E7 synthetic long peptide vaccine; CUE-1 is a fusion protein nased on an E7 specific HLA * 02 restricted TCR expressed in T cells expanded ex vivo and adoptively transferred. Chemotherapeutic drugs: paclitaxel, carboplatin, cisplatin, and docetaxel.

## Data Availability

Not applicable.
